# 转录因子Snail与肿瘤的上皮细胞间质化

**DOI:** 10.3779/j.issn.1009-3419.2011.09.12

**Published:** 2011-09-20

**Authors:** 志浩 吴

**Affiliations:** 300052 天津，天津医科大学总医院，天津市肺癌研究所，天津市肺癌转移与肿瘤微环境实验室 Tianjin Key Laboratory of Lung Cancer Metastasis and Tumor Microenviroment, Tianjin Lung Cancer Institute, Tianjin Medical University General Hospital, Tianjin 300052, China

肿瘤对人体的危害极大，其引起的死亡大多是因为肿瘤转移。肿瘤的转移是一个十分复杂的过程，它是多种基因参与的结果，不仅仅是遗传性状的改变，还包括细胞与细胞之间、细胞与细胞外基质之间的相互作用。一些在胚胎发育中起着重要作用的基因常常在癌组织中重新激活，提示其可能有促进肿瘤发生和/或转移的作用。肿瘤的浸润和转移是通过细胞粘附分子的改变而实现的，这些粘附分子也是许多致癌因素最终的作用位点。而上皮细胞间质化（epithelial-mesenchymal transition, EMT）与肿瘤细胞的原位侵袭和远处转移有着密切的关系^[1, 2]^。因此对于细胞粘附分子在EMT中的作用一直是国内外研究的热点。这对于揭示肿瘤发生发展的细胞分子生物学机制有重要的意义，同时也开拓了思路，为肿瘤治疗的新方法、新途径提供了有力的依据。

## 1 EMT的概念

EMT是指具有极性的上皮细胞转换成为具有活动能力的间质细胞并获得侵袭和迁移能力的过程，最早被用于描述胚胎发育中某些特定部位的上皮细胞发生的形态学改变，如中胚层以及神经嵴的形成等。它存在于人体多个生理和病理过程中。肿瘤发生EMT时，上皮细胞表型发生可逆的改变，上皮细胞失去了细胞与细胞之间正常的结构，包括上皮细胞极性的丧失和间质特性（成纤维细胞样的外形、波形蛋白等基因的表达）的获得，细胞之间彼此分离，获得活动能力并抵抗凋亡。最近，有种类似的转化也被归类到EMT中，称为不完全EMT，常见于伤口愈合或血管生成^[3]^，细胞既具有一些上皮细胞的特性，也表现部分间质细胞的特征。其与完全EMT不同的是，细胞之间的粘附位点和桥粒尽管较为稀疏但仍然存在，细胞骨架改变但仍然表达角蛋白，具有粘附力的同批细胞同步移行^[3]^。在肿瘤的侵袭与转移中，EMT和不完全EMT过程均可发生。

## 2 转录因子Snail的分子结构和功能

Snail是近年发现的锌指转录因子，属于转录因子中的Snail超家族成员。其第一次是在果蝇中被描述，Snail突变的果蝇胚胎表现为原肠胚缺陷，经证实与E-cadherin下调有关^[4]^。Snail家族成员包括Snail 1（snail）、Snail 2（slug）和Snail 3（smuc），均在脊椎动物胚层形成中起重要的作用^[5]^。在胚胎发育中，Larue等^[6]^描述在Snail的刺激下上皮细胞可向间充质细胞转化并形成中胚层和神经嵴。

Snail家族成员在结构上具有很高的相似性，由一个高度保守的羧基末端和一个高度可变的氨基末端构成，各成员间结构上的不同主要表现在中间P-S富集区。其中C-末端包含4个-6个C_2_H_2_型的锌指DNA结合区域，可以与含有E-box（CAGGAG）的启动子序列结合，每个锌指包含两个β链和一个α螺旋，α螺旋的氨基末端结合在DNA大沟上，两个保守的C_2_H_2_与锌离子协同作用^[5]^。

Cheng等^[7]^分析了乳腺导管癌中E-cadherin失活的不同机制，提出了Snail在人原位癌中表达并确定了Snail的表达与E-cadherin的减少或缺失有关。Snail氨基末端的SNAG反式激活区域（Snail Gfi）是Snail的转录抑制作用关键部位。Comijn等^[8]^研究发现，切除SNAG区域的Snail蛋白没有抑制E-cadherin启动子的活性，表明SNAG区域对于Snail抑制E-cadherin启动子的活性是必不可少的。Snail通过Sin3A^[9]^介导，与组蛋白脱乙酰酶HDAC1和2形成抑制复合物来下调E-cadherin的表达。在果蝇中Snail没有SNAG结构域但含有PxDLSx序列，与协同抑制物CtBP相互作用来抑制基因表达^[5, 10]^。

Snail通过抑制E-cadherin不仅可以下调上皮细胞标记蛋白的表达，同时也促进间质细胞标记蛋白的表达；而E-cadherin过表达可以反过来抑制Snail对间质细胞标记蛋白的诱导^[11]^。但这些间质细胞标记蛋白的激活并不完全是由E-cadherin降低引起的，在E-cadherin表达缺陷的细胞中Snail也可以促进间质细胞标记蛋白的表达。有研究^[12]^表明，Snail在胞核中与β-catenin相互作用，可激活Wnt通路的靶基因，说明在某些情况下，Snail可以直接作为激活分子发挥作用。

Snail蛋白的中央区域起着调节其稳定性和定位的作用，在这一区域内有许多磷酸化位点。GSK-3β可以磷酸化Snail的Ser104和107位点，从而暴露出位于氨基酸132-143位点之间的出核转运序列来促进Snail的出核。在胞浆中Snail被GSK-3β进一步磷酸化后^[13]^，与β-TrCP1泛素连接酶结合，导致其泛素化和降解^[14]^。这种磷酸化可以被SCP（small C-terminal domain phosphatase）抑制，其可以使Snail稳定在胞核内^[15]^。Snail也可以通过磷酸化其它位点来调节其活性。蛋白激酶A和CK2分别磷酸化Snail的Ser11和92位点来加强对E-cadherin的抑制和与Sin3A协同抑制物的相互作用^[16]^。PAK1可以磷酸化Snail的C-末端使其停留在细胞核内^[17]^。除了β-TrCP1，Snail还可以与另外一种E3泛素连接酶-FBXL14相互作用，促使其泛素化和降解^[18]^。FBXL14与Snail中央区域的氨基酸120-151位点相互作用，与β-TrCP1不同的是，它们的结合并不是依赖前面提到的磷酸化，但泛素连接酶却都是通过Lys138和146发挥作用的。

## 3 转录因子Snail与钙粘蛋白E-cadherin的相关性

E-cadherin是钙黏附素中的重要成员，也称为L-CAM、CAM120/80或Arc-1，是Ca^2+^依赖性跨膜糖蛋白，分为细胞外区、跨膜区和细胞内区。其细胞外区的组-丙-缬（HAV）序列为识别位点，以此来识别和介导同种细胞间的粘附反应^[19]^。E-cadherin普遍存在于各类上皮细胞中，在维持正常的细胞形态、细胞极性和组织结构完整性中起着重要的作用^[20]^。在正常的上皮细胞中，E-cadherin稳定表达，但在多种上皮性肿瘤中其表达下调^[21]^。E-cadherin表达的减少或丧失，使细胞间的粘附力减弱，肿瘤细胞易于从原发灶上脱落。而肿瘤细胞从原发部位脱落是肿瘤浸润和转移的第一步^[22]^。

E-cadherin被认为是Snail直接作用的靶基因，Snail主要以锌指区域与E-cadherin启动子上的E-box序列结合，达到抑制E-cadherin转录的作用^[2]^。有研究^[23]^表明，在正常的黑色素细胞中E-cadherin高表达而Snail低表达，但在各种黑色素细胞瘤细胞系中，Snail表达明显升高而E-cadherin明显降低。Olmeda等^[24]^证实，通过RNA干扰技术来沉默MOCK-Snail细胞中Snail的表达，可以引起E-cadherin表达明显的增高。由此可知，Snail表达的升高与E-cadherin表达的降低密切相关。

## 4 Snail对EMT的作用及与肿瘤转移的关系

在胚胎的中胚层及神经嵴的发育中，Snail是必不可少的，它触发了细胞的EMT，促使细胞从原条和神经管分层以及随后的移动^[6]^。在脉络膜新血管生成中，Hirasawa等^[25]^也证明了Snail是视网膜色素细胞EMT的主要调控因子。EMT是肿瘤发展的决定因素，E-cadherin的表达降低或缺失是EMT发生的标志，而Snail通过抑制E-cadherin的表达来促进EMT的发生。Snail在浸润性乳腺癌的上皮及内皮细胞中过表达，而在正常乳腺组织的上皮及内皮细胞中不表达^[26, 27]^。在对鳞状细胞癌的研究^[28]^中发现，E-cadherin表达阴性的克隆细胞中Snail高表达且侵袭能力强，E-cadherin表达阳性的克隆细胞几乎检测不到Snail的表达且侵袭力弱。E-cadherin阴性的鳞状癌细胞表现为间皮细胞的形态特征，同时与E-cadherin阳性细胞相比，体外侵袭力增强^[29]^。将Snail转染到E-cadherin阳性的肝癌细胞Li-7后，E-cadherin的表达丧失且细胞的侵袭性增强^[30]^。利用RNA干扰技术来沉默细胞中的Snail表达，不仅可以引起E-cadherin表达的增加，同时可以使细胞的侵袭力减弱^[24]^。Natsugoe等^[31]^通过对194例食管鳞状上皮癌病例进行分析，发现相对于E-cadherin表达较高的细胞，E-cadherin表达降低的细胞侵袭力增加并有更多的淋巴结转移和淋巴侵袭；而Snail表达阳性的细胞比Snail阴性的细胞侵袭能力更强，累及更多的淋巴组织。E-cadherin低表达或Snail高表达的患者临床表现也较差，且总生存率明显低于E-cadherin高表达或Snail低表达的患者。在非小细胞肺癌模型的体内及体外实验也证实了Snail可以诱导EMT^[32]^。近年来，研究^[33]^表明Snail也可以通过调节其它因子来促进肿瘤的发展。在结肠癌中，Snail通过上调IL-8及其它基因，诱导肿瘤干细胞的活性。在前列腺癌中Snail通过调节氧化应激酶来促进ROS诱导的EMT，从而促进肿瘤的发展^[34]^。

## 5 小结

Snail在上皮细胞中过表达使得细胞向成纤维细胞转化，同时E-cadherin表达降低，肿瘤发生并获得侵袭性和转移性。近年来，Snail在肿瘤转移中的作用越来越受到关注。揭示Snail的功能和作用机制，不仅可以明确肿瘤的发生和转移机制，同时也为肿瘤的治疗和预防提供了新的思路。但是目前对Snail的研究仍有许多问题不甚明确：Snail在抑制E-cadherin表达、诱导EMT时，这些细胞因子是怎样协同作用的，通过哪些信号通路，这些细胞因子是否也调控其它基因的表达来促进EMT，Snail是否可以直接调控其它因子来促进EMT及其机制等。这些复杂的问题需要我们进一步的探讨和研究，希望能够为上皮肿瘤的治疗和预防提供更有利的依据。
